# Goat lung surfactant for treatment of respiratory distress syndrome among preterm neonates: a multi-site randomized non-inferiority trial

**DOI:** 10.1038/s41372-019-0472-0

**Published:** 2019-09-04

**Authors:** Kajal Jain, Sushma Nangia, Vishnu Bhat Ballambattu, Venkataseshan Sundaram, M. Jeeva Sankar, Siddharth Ramji, Sreenivas Vishnubhatla, Anu Thukral, Yogendra Kumar Gupta, Nishad Plakkal, Mangalabharathi Sundaram, Mamta Jajoo, Praveen Kumar, Kumutha Jayaraman, Ashish Jain, Arvind Saili, Anitha Murugesan, Deepak Chawla, Srinivas Murki, Ruchi Nanavati, Suman Rao, Umesh Vaidya, Ashish Mehta, Kamal Arora, Jayashree Mondkar, Sugandha Arya, Monika Bahl, Alpana Utture, Swati Manerkar, Swarna Rekha Bhat, Tushar Parikh, Manish Kumar, Anurag Bajpai, Sindhu Sivanandan, Pawandeep Kaur Dhawan, Gayatri Vishwakarma, Sudhakar Bangera, Sumit Kumar, Shridhar Gopalakrishnan, Atul Jindal, Chandra Kumar Natarajan, Anumeet Saini, Sukanya Karunanidhi, Meenakshi Malik, Parul Narang, Gurkirat Kaur, Chander Prakash Yadav, Ashok Deorari, Vinod K. Paul, Ramesh Agarwal

**Affiliations:** 1 All India Institute of Medical Sciences (AIIMS), New Delhi, India; 2 Lady Hardinge Medical College (LHMC), New Delhi, India; 3 Jawaharlal Institute of Postgraduate Medical Education and Research (JIPMER), Puducherry, India; 4 Postgraduate Institute of Medical Education and Research (PGIMER), Chandigarh, India; 5 Maulana Azad Medical College (MAMC), New Delhi, India; 6 Translational Health Sciences and Technology Institute, Faridabad, India; 7 Institute for Child Health (ICH), Chennai, India; 8 Chacha Nehru Bal Chikitsalaya (CNBC), New Delhi, India; 9 Government Medical College & Hospital, Chandigarh, India; 10 Fernandez Hospital, Hyderabad, India; 11 King Edward Memorial Hospital, Mumbai, India; 12 St. John’s Medical College, Bangalore, India; 13 King Edward Memorial Hospital, Pune, India; 14 Arpan Newborn Care center and Sterling NICU, Ahmedabad, India; 15 Dayanand Medical College and Hospital, Punjab, India; 16 Lokmanya Tilak Municipal General Hospital (SION Hospital), Mumbai, India; 17 Safdarjung Hospital, New Delhi, India; 18 Clinical Development Services Agency, Faridabad, India; 19 Command Hospital, Panchkula, India; 20 All India Institute of Medical Sciences (AIIMS), Raipur, India; 21 Cloudnine Hospital, Chennai, India; 22 National Institute of Malaria Research, New Delhi, India; 23 National Institution for Transforming India (NITI Aayog), Government of India, New Delhi, India

## Abstract

**Objective:**

To investigate the safety and efficacy of goat lung surfactant extract (GLSE) compared with bovine surfactant extract (beractant; Survanta®, AbbVie, USA) for the treatment of neonatal respiratory distress syndrome (RDS).

**Study design:**

We conducted a double-blind, non-inferiority, randomized trial in seven Indian centers between June 22, 2016 and January 11, 2018. Preterm neonates of 26 to 32 weeks gestation with clinical diagnosis of RDS were randomized to receive either GLSE or beractant. Repeat dose, if required, was open-label beractant in both the groups. The primary outcome was a composite of death or bronchopulmonary dysplasia (BPD) at 36 weeks postmenstrual age (PMA). Interim analyses were done by an independent data and safety monitoring board (DSMB).

**Result:**

After the first interim analyses on 5% enrolment, the “need for repeat dose(s) of surfactant” was added as an additional primary outcome and enrolment restricted to intramural births at five of the seven participating centers. Following second interim analysis after 98 (10% of 900 planned) neonates were enroled, DSMB recommended closure of study in view of inferior efficacy of GLSE in comparison to beractant. There was no significant difference in the primary outcome of death or BPD between GLSE group (*n* = 52) and beractant group (*n* = 46) (50.0 vs. 39.1%; OR 1.5; 95% CI 0.7–3.5; *p* = 0.28). The need for repeat dose of surfactant was significantly higher in GLSE group (65.4 vs. 17.4%; OR 9.0; 95% CI 3.5–23.3; *p* < 0.001).

**Conclusions:**

Goat lung surfactant was less efficacious than beractant (Survanta®) for treatment of RDS in preterm infants. Reasons to ascertain inferior efficacy of goat lung surfactant requires investigation and possible mitigating strategies in order to develop a low-cost and effective surfactant.

## Introduction

Surfactant replacement therapy (SRT) forms the cornerstone of management of moderate to severe respiratory distress syndrome (RDS) among preterm neonates and it is associated with reduced mortality and pulmonary air leak [1, 2]. These benefits have been confirmed through a meta-analysis conducted to study the efficacy and safety of SRT for preterm neonates in low- and middle-income countries (LMICs) [3]. Surfactant therapy is cost effective [4] and is included in the World Health Organization’s Essential drug list [5]. However the high cost of surfactant has precluded its optimum access in LMICs where 90% of global burden of neonatal deaths occur due to complications of prematurity. Given India’s proven track record of producing high-quality drugs at low cost, development of indigenous surfactant has been listed as an important priority by the Government of India [6].

Natural surfactants used today are sourced from bovine or porcine origin and extracted from minced lung tissue or by lung lavage. Researchers at All India Institute of Medical Sciences (AIIMS), New Delhi, prepared surfactant from slaughtered goat lungs in 1998 by chloroform–methanol extraction method [7]. Subsequently, an Indian pharmaceutical company (Cadisurf®; Cadila Pharmaceuticals, Ahmedabad, India) carried out product development further. The goat lung surfactant extract (GLSE) contains phospholipids (25 mg/mL phospholipids) and surfactant-associated proteins (<1%). The product underwent independent biochemical standardization by the manufacturer and was found to be identical in composition to beractant (Survanta; AbbVie, USA) with regard to its phospholipid and peptide content. Its biological activity as a surface-active agent was documented in in-vitro studies and in a rat lung model of RDS. The product was shown to be microbiologically sterile and no adverse events were documented in animal toxicity studies performed in compliance with Indian regulatory guidelines. The cost of SRT using GLSE was estimated to be less than a tenth of imported surfactants. The product, however, awaited clinical validation. The objective of this study was to compare the safety and efficacy of GLSE with that of the standard bovine lung surfactant (beractant; Survanta®) in the treatment of RDS.

## Methodology

### Study design

In this multicenter, randomized, double-blind, parallel group, non-inferiority trial, we compared the safety and efficacy of GLSE (Cadisurf®, Cadila Pharmaceuticals, Ahmedabad, India) with beractant (Survanta®, AbbVie, USA) for the treatment of RDS in preterm neonates. The trial was undertaken at seven tertiary care academic centers in India from June 22, 2016 to January 11, 2018. Five of the participating NICUs were intramural (inborn) units, while the other two exclusively cared for extramural (outborn) neonates. The study protocol was approved by the institutional ethics committee (IEC) of each participating center. The trial was registered with the Clinical Trials Registry - India (CTRI/2015/07/005968) and ClinicalTrials.gov (NCT02774044).

### Participants

Preterm neonates born at 26–32 weeks’ gestation and admitted to any of the participating NICUs were eligible for enrolment if they developed clinical features of RDS (fast breathing or chest retractions) within 6 h of birth and fulfilled criteria to receive surfactant therapy within 24 h of birth. The criteria for SRT were any one of the two: (i) FiO_2_ requirement 40% or higher while on continuous positive airway pressure (CPAP) to maintain oxygen saturation between 90 and 95%, (ii) need for intubation and mechanical ventilation because of CPAP failure or severe respiratory distress. Chest X-ray was not mandated prior to SRT. Exclusion criteria were (i) significant perinatal asphyxia, defined as need for chest compressions during resuscitation or metabolic acidosis (pH < 7.0) in umbilical arterial blood gas or arterial blood gas obtained within 1 h of life, (ii) major congenital anomalies or known chromosomal aberrations, (iii) receipt of prophylactic surfactant, (iv) clinical evidence of pulmonary air leak or pulmonary hemorrhage prior to enrolment, and (v) shock requiring vasopressor support prior to enrolment. Parents of potentially eligible neonates were approached both before and after birth, and written informed consent was obtained from parents or legally acceptable representative prior to enrolment.

### Randomization

Neonates were randomly assigned to receive GLSE or beractant. Randomization lists were prepared by an independent statistician using SAS 9.4 software (SAS Institute Inc., USA). Assignments were stratified according to center and gestational age (26–27 weeks and 28–32 weeks). Within each stratum, a 1:1 allocation ratio and block randomization with variable block sizes (4 to 10) were used. Neonates of multiple births with more than one eligible participant were randomized individually.

### Intervention and blinding

GLSE was supplied in single-use glass vials containing 8 mL (200 mg phospholipids; 25 mg/mL) of the product suspended in preservative-free 0.9% sodium chloride solution. Beractant (Survanta, AbbVie, USA) was commercially procured. Since the two surfactant products differed in color, consistency, and packaging, an independent team at a central location repacked the vials in identical sealed cardboard boxes and serially labeled them based on the allocation sequence for two gestation strata. They were then transported to study centers while maintaining the cold chain.

The research team, parents, data analysis team, monitoring team, and data and safety monitoring board (DSMB) were blinded to the intervention. To ensure blinding of physicians and nurses who administered the surfactant, we employed an innovative method *(SOP for blinding provided in* Supplementary information*)*. A team of nurses (IP nurses) from the ward adjoining NICU were trained to load surfactant as per the study blinding protocol. On enrolment, the research team summoned an IP nurse who opened the sealed box as per the serial number, drew up the specified amount of surfactant in a specially manufactured opaque syringe, connected an opaque catheter to its nozzle and handed it over to the clinical team at bedside ready for administration. The surfactant loading was performed in a cordoned area of the NICU. After surfactant administration, the IP nurse collected the empty syringe and catheter from the clinicians and sealed all the used material and vial in a bag to be retrieved by the nodal team later.

### Procedures

Surfactant was administered via an opaque catheter passed through the endotracheal tube in four equal aliquots. Post surfactant, neonates were extubated to CPAP (intubation, surfactant administration, and extubation; InSurE) if they had adequate spontaneous respiratory efforts and were hemodynamically stable; else, they were continued on mechanical ventilation and weaned appropriately. Neonates were eligible for repeat dose(s) of surfactant (maximum of three additional doses) at an interval of 4–6 h if they were still intubated, or if oxygen requirement was 40% or more on nasal CPAP, or at the discretion of the treating physician. For repeat doses in both the groups, open-label beractant (Survanta) was used. Participating NICUs were provided with standard operating procedures (SOP) for the management of neonatal morbidities; however, they were permitted to follow unit protocol, whenever appropriate. Participants were followed up until 36 weeks of postmenstrual age (PMA) or death.

### Study outcomes

The primary outcome of the study was a composite of death or bronchopulmonary dysplasia (BPD) defined physiologically at 36 weeks PMA as requirement of supplemental oxygen of 30% or more or the need for positive-pressure support to achieve oxygen saturation  greater than 90% [8]. Infants requiring less than 30% oxygen were labeled to have BPD if they failed an oxygen challenge test [9] done by study personnel masked to treatment assignments. After the first interim analysis, DSMB added need for repeat dose of surfactant as an additional primary outcome. Secondary outcome measures included area under the curve (AUC) for fractional inspired oxygen (FiO_2_) in the first 48 h post-surfactant, type of respiratory support needed at 72 h and 7 days of age, occurrence of pulmonary hemorrhage within 48 h of SRT, clinically important air leak within 72 h of SRT, grade 3 or 4 intraventricular hemorrhage (IVH), periventricular leukomalacia (PVL)—cystic and noncystic, sepsis in the first week of life, retinopathy of prematurity (ROP) requiring treatment, mortality within 28 days of life, days on respiratory support (mechanical ventilation and CPAP), and duration of hospital stay. The various study outcomes and their definitions are provided in Supplementary information. Area under curve of FiO_2_  provided a comprehensive summary of the hourly oxygen requirements in the first 48 h after administration of surfactant. The curve was plotted for each neonate and the mean for all neonates was calculated for single summary index.

### Monitoring of study

Quality assurance and quality control systems were established to ensure that trial conduct, data generation, data recording, and reporting were done in compliance with the protocol, good clinical practice standards and applicable regulatory requirements. All investigators and site teams were trained on the study protocol, trial related procedures, information pertaining to investigational products, procedure for reporting serious adverse events (SAEs), and SOPs for completing case record forms (CRF). The study team maintained adequate and accurate records using site file, source documents and logs designed to capture clinical data. All study data were first captured manually in CRF and then transcribed onto the electronic CRF at sites. (Datasets are available upon request from the corresponding author.) Site users were trained on Accelient study database (*Trianz, Acceliant eClinical Suite, Bengaluru*). Site monitoring visits were undertaken on regular basis and information recorded in the CRF was verified against source documents. Discrepancies observed during quality checks were flagged for the site investigators to verify and reconcile. Death and other SAEs were reported to the regulatory authorities and causality assessment was carried out. The DSMB monitored the progress of study and safety data for prespecified outcomes including death and other SAEs. The study had pre-specified safety stopping bounds and interim analyses were planned after 5, 33, and 66% enrolment of subjects.

### Sample size

Based on the retrospective data for 1 year provided by the participating centers, the risk of death or BPD among inborn preterm neonates ≤32 weeks gestation was estimated to be 40%. The sample size was computed considering a non-inferiority margin for GLSE of 10% higher than this baseline rate. For an alpha error of 5% and power of 90%, the sample size required was 412 infants per group and we increased the sample size to 450 per group (total sample of 900) to account for a 10% drop out rate after enrolment.

### Statistical analysis

All data were analyzed according to intention-to-treat principle using Stata software (version 14.2). Continuous data were summarized using mean and standard deviation or median and interquartile range, as appropriate. Categorical data were summarized using frequency and percentage. Student’s unpaired *t* test or Wilcoxon Rank Sum test was used for comparison of continuous data and Chi-square or Fisher’s exact test was applied for comparison of categorical data. Multivariable logistic regression analysis was carried out for the outcome variables after adjustment for study center and baseline characteristics that were substantially different between the two groups (>10% difference) on univariate analysis. Risk difference along with 95% CI was calculated to assess the non-inferiority.

## Results

The first interim safety analysis was conducted after the enrolment of initial 5% of neonates (45 neonates with 42 enroled at five inborn centers). The DSMB analyzed the data by groups A and B while still being blinded to the nature of intervention. There was no difference in death or BPD but the need for repeat dose of surfactant was different between the two groups. Because the two groups differed in baseline characteristics, no definite conclusions could be made. The DSMB recommended to restrict enrolment to inborn neonates, to add ‘need for repeat dose of surfactant’ as an additional primary outcome, and to conduct an additional interim analysis after completing 10% enrolment. In the second interim analysis after 98 inborn neonates completed follow up, the DSMB recommended that the study be closed to further enrolment in view of poor efficacy of GLSE in comparison with beractant. The recruitment status at different sites at the time of study closure is provided in Supplementary information.

**Fig. 1:**
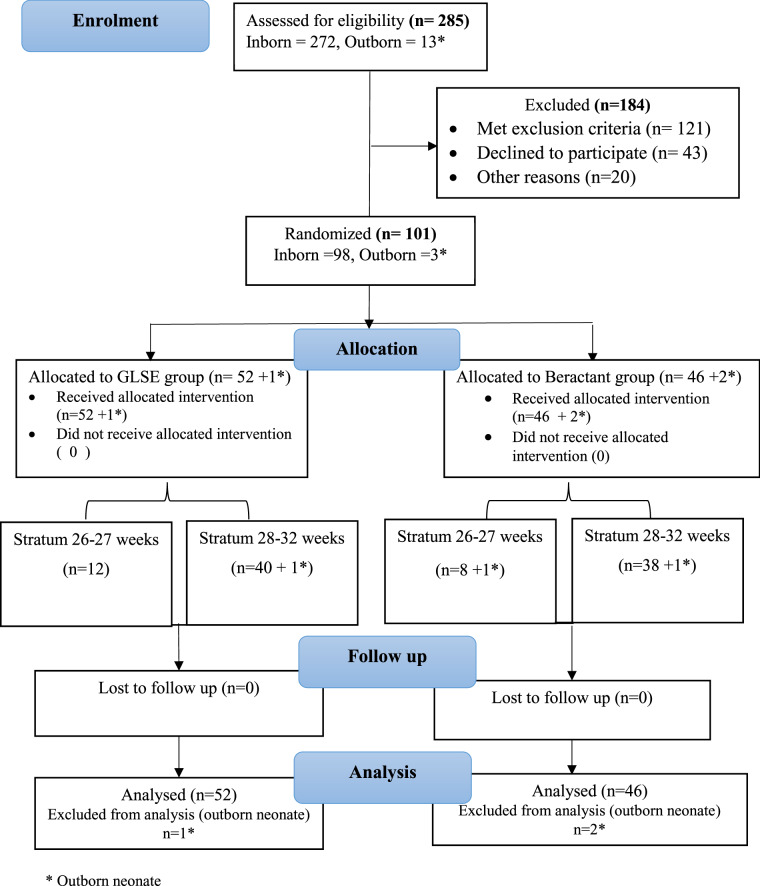
Participant flow

At the time of study closure, 285 preterm neonates ≤32 weeks gestation had been assessed for eligibility, and 101 from seven (five inborn and two outborn) centers were randomized to either of the groups. As directed by DSMB, outborn neonates (*n* = 3) were not included in the final analysis. Thus 98 inborn neonates randomized to the GLSE group (*n* = 52) or the beractant group (*n* = 46) were analyzed (Fig. 1). The baseline characteristics were comparable between the two groups except a higher percentage of male neonates enroled in the GLSE group (78.3 vs. 48.1%; *p* = 0.002) (Table 1). The median age at eligibility and receipt of surfactant were similar, with most neonates receiving surfactant within 2 h of being eligible in both the groups.

**Table 1:** Baseline characteristics of the enroled infants

Characteristic	GLSE (*n* = 52)	Beractant (*n* = 46)	*p*-value
Maternal			
Antibiotics in 7 days before delivery	18 (34.6)	18 (39.1)	0.64
Gestational diabetes	6 (11.5)	3 (6.5)	0.39
Antenatal steroids			
None/unknown	8 (15.4)	8 (17.4)	
Incomplete	17 (32.7)	18 (39.1)	0.70
Complete	27 (51.9)	20 (43.5)	
Clinical chorioamnionitis	(*n* = 47)	(*n* = 43)	0.35
	3 (6.4)	1 (2.3)	
Rupture of membranes for ≥18 h	16 (30.8)	8 (17.4)	0.12
Cesarean delivery	23 (44.2)	18 (39.1)	0.61
Antepartum hemorrhage	14 (26.9)	7 (15.2)	0.16
Neonatal			
Gestation in weeks	29.2 ± 1.9	29.2 ± 1.8	0.84
Birth weight, g	1192 ± 312	1156 ± 298	0.56
Small for gestational age	6 (11.5)	5 (10.9)	0.63
Male gender	25 (48.1)	36 (78.3)	0.002
Multiple births	13 (25.0)	12 (26.1)	0.90
Positive pressure ventilation (PPV) at birth	32 (61.5)	22 (47.8)	0.17
Delayed cord clamping	6 (11.5)	6 (13.1)	0.82
Delivery room CPAP	22 (42.3)	24 (52.2)	0.33
Median age at eligibility, h	1.0 (0.2, 2)	1.5 (1, 3.8)	0.82
Median age at surfactant administration, h	2.4 (1.7, 4.5)	2.4 (1.8, 4.7)	0.61
InSurE^a^	29 (55.8)	32 (69.6)	0.16

Data expressed in *n* (%), mean ± SD, or median (IQR)

^a^Intubation–surfactant–extubation within 30 min of completion of surfactant administration

There was no significant difference in the primary outcome of death or BPD between the GLSE and beractant groups (50.0 vs. 39.1%; OR 1.5; 95% CI 0.7–3.5; *p* = 0.28). The need for repeat dose of surfactant was higher in the GLSE group (65.4 vs. 17.4%; OR 9.0; 95% CI 3.5–23.3; *p* < 0.001) (Table 2).The mean number of surfactant doses in the GLSE group was 1.9 ± 0.8 compared with 1.2 ± 0.6 in the beractant group (*p* = 0.001) (Table 3).

**Table 2:** Primary outcomes and the components of the composite primary outcome

Outcome	GLSE group (*n* = 52)	Beractant (*n* = 46)	OR (95% CI)	Risk difference Beractant−GLSE (95% CI)
Death or BPD at 36 weeks PMA	26 (50.0)	18 (39.1)	1.5 (0.7–3.5)	−10.8 (−30.5, 8.7)
Repeat dose of surfactant	34 (65.4)	8 (17.4)	9.0 (3.5–23.3)	−48.0 (−64.9, −31.0)
Death	21 (40.4)	14 (30.4)	1.5 (0.7–3.6)	−9.94 (−28.7, 8.9)
BPD	5/31 (16.1)	4/32 (12.5)	1.6 (0.6–4.9)	−3.6 (−20.9, 13.7)

*BPD* bronchopulmonary dysplasia, *PMA* postmenstrual age, *OR* odds ratio

**Table 3:** Surfactant doses received in GLSE and Beractant groups

Characteristic	GLSE (*n* = 52)	Beractant (*n* = 46)	*p*-value
No. of surfactant doses received (including the first IP dose)			
1	18 (34.6)	38 (82.6)	
2	19 (36.5)	7 (15.2)	<0.001
≥3	15 (28.8)	1 (2.2)	
Mean number of surfactant doses received	1.94 ± 0.8	1.22 ± 0.6	<0.001
Age at second dose (h)	(*n* = 34)	(*n* = 8)	
Mean ± SD	11.5 ± 5.8	13.6 ± 5.4	0.36
Median (IQR)	9.8 (7.8,13.4)	12.2 (710,16.6)	
Age at third dose (h)	(*n* = 15)	(*n* = 1)	–
Mean ± SD	29.0 ± 19.7	19.6	
Median (IQR)	17.6 (13,47.2)	19.6	
Mean age at fourth dose (h)	0	(*n* = 1) 28.2	–

*IP* Investigational product

Among secondary outcomes, average FiO_2_ required during first 48 h post-surfactant administration or until death (if died within 48 h) was higher in GLSE group (50.5 ± 21.2 vs. 33.3% ± 14.5; 17.2%, 95% CI 9.8–24.6, *p* < 0.001). The duration of mechanical ventilation in first 48 h postsurfactant administration was also higher in GLSE group (28.5 vs. 16.0 h; mean difference 12.5; 95% CI 4.8–20.1, *p* = 0.001) (Table 4). Other secondary outcomes and SAEs including pulmonary hemorrhage within 48 h of surfactant administration, air leak within 72 h of surfactant administration, sepsis in first 7 days after birth, PIVH, necrotizing enterocolitis, hemodynamically significant patent ductus arteriosus (PDA), BPD, ROP requiring treatment, duration of NICU stay and hospital stay were comparable between the groups (Table 4). No case of PVL was noted in either group.

**Table 4:** Secondary outcomes

Secondary outcome	GLSE (*n* = 52)	Beractant (*n* = 46)	OR/mean difference (95% CI)	*p*-value
Area under curve (AUC) for FiO_2_ 0–48 h of surfactant administration	2055 ± 793	1511 ± 697	543 (242 to 845)	0.005
Average FiO_2_ (%) required during first 48 h or until death after surfactant administration^a^	50.5 ± 21.2	33.3 ± 14.5	17.2 (9.8 to 24.6)	<0.001
Cumulative duration of mechanical ventilation in first 48 h after surfactant administration	28.5 ± 19.1	16 ± 19	12.5 (4.8 to 20.1)	0.002
Pulmonary hemorrhage within 48 h of surfactant administration	5 (9.6)	3 (6.5)	1.5 (0.3–6.7)	0.58
Air leak within 72 h of surfactant administration	3 (5.8)	0	–	–
Sepsis in 7 days				
Culture positive	6 (11.5)	2 (4.4)	1.7 (0.9–2.5)	
Culture negative	8 (15.4)	5 (10.9)	2.3 (1.4–3.2)	0.18
No sepsis	38 (73.1)	39 (84.8)		
Intraventricular hemorrhage (IVH)	9 (17.3)	3 (6.5)	3 (0.8–11.8)	0.12
Necrotizing enterocolitis stage ≥2	1 (1.9)	1 (2.2)	0.9 (0.1–14.5)	0.93
Hemodynamically significant PDA	10 (19.2)	10 (21.7)	0.8 (0.3–2.3)	0.76
Cystic periventricular leukomalacia (PVL)	0	0	–	–
ROP requiring laser/surgery/VEGF inhibitor	3 (5.8)	1 (2.2)	2.8 (0.3–27.4)	0.39
BPD	*n* = 31	*n* = 32		
Moderate	3 (9.7)	2 (6.2)		
Severe	2 (6.4)	2 (6.2)	1.6 (0.6–4.9)	0.81
NICU stay (days)	27.7 ± 29.2	28.6 ± 22.5	0.9 (−9.6 to 11.5)	0.86
Hospital stay (days)	31.6 ± 32.0	31.7 ± 21.9	0.1 (−11.0 to 11.3)	0.98
No of participants with				
No SAE	11 (21.2)	18 (39.1)	1.0	
At least 1 SAE other than death	20 (38.5)	14 (30.4)	2.33 (0.76–7.3)	0.09
>1 SAE including death	29 (55.7)	21 (45.7)	2.25 (0.8–6.4)	0.08

Data expressed in *n/N* (%) or mean ± SD or median (IQR)

*SAE* serious adverse event, *VEGF* vascular endothelial growth factor

^a^Recorded on hourly basis

**Fig. 2:**
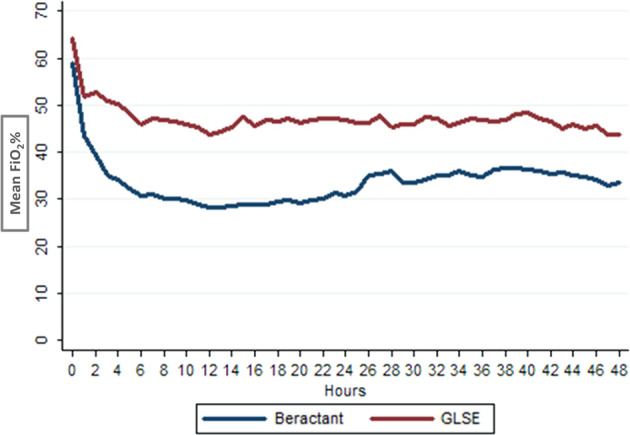
Mean FiO_2_ values from 1 to 48 h postsurfactant in GLSE and Beractant groups

A multivariable analysis of outcomes was performed adjusting for study center and baseline characteristics that were substantially different (>10%; even if statistically not significant) between two groups namely, gender, rupture of membranes for more than 18 h, antepartum hemorrhage, need for positive pressure ventilation after birth, use of delivery room CPAP and InSurE. There was no difference in mortality between the groups (*p* = 0.82) but need for the repeat dose of surfactant (Table 4), need for mechanical ventilation within 24 h and average FiO_2_ required in first 48 h after surfactant administration (Fig. 2) was significantly higher in the GLSE group compared with beractant group (Table 5).

**Table 5:** Multivariable analysis of key outcomes

Outcome	GLSE *n* = 52 n (%)	Beractant *n* = 46 n (%)	Unadjusted odds ratio (95% CI), *p* value	Adjusted odds ratio^a^ (95% CI), *p*-value
Death	21 (40.4%)	14 (30.4%)	1.5 (0.7–3.6) *p* = 0.306	1.13 (0.40–3.29) *p* = 0.815
Repeat dose (one or more)	34 (65.4%)	8 (17.4%)	9.0 (3.5–23.3) *p* < 0.001	9.8 (3.2 –30.1) *p* < 0.001
Need for mechanical ventilation at 24 h after surfactant	32/49 (65.3%)	15/46 (32.6%)	3.89 (1.66–9.12) *p* = 0.002	5.1 (1.5 –17.2) *p* = 0.009
Average FiO_2_ required in first 48 h after surfactant administration (%)	50.5 ± 21.2	33.3 ± 14.5	Difference in means 17.2 (9.8–24.6) *p* < 0.001	Difference in means 12.5 (5.5–19.6) *p* < 0.001

*FiO*_2_-fractional inspired oxygen concentration

^a^Adjusted for study center and baseline characteristics that were substantially different (10%) between group A and group B: sex of baby, rupture of membranes for more than 18 h, antepartum hemorrhage, required positive pressure ventilation immediately after birth, receipt of delivery room CPAP, and InSurE (intubation, surfactant administration and extubation)

## Discussion

The GLSE trial is the first to evaluate an indigenous low-cost surfactant extracted from goat lungs for neonatal RDS. The study was prematurely terminated after enrolment of 10% of planned sample size in view of poor efficacy of GLSE when compared with beractant. While the composite primary outcome of death or BPD was not different between the two groups, the need for repeat doses of open-label surfactant was higher in the GLSE group. Other objective pointers of poor efficacy included the higher need for mechanical ventilation within 24 h and higher average FiO_2_ required in first 48 h post-surfactant administration in the GLSE group.

The high cost of surfactant therapy continues to be a major barrier in LMIC countries. The demand for an indigenous surfactant that is efficacious and safe yet cheap remains a top research priority in these countries [6]. The *Instituto Butantan* in Brazil developed a low-cost porcine surfactant using a new technology that involved macerating swine lung followed by organic extraction and cellulose adsorption [10]. A multicenter RCT in Brazil randomized 327 infants to receive Butantan or standard surfactants (Survanta or Curosurf). There was no difference in the primary outcome of mortality at 72 h of life (14.1 vs. 14.1%; *p* = 0.98) or on day 28 of life (39.8 vs. 33.3%; *p* = 0.24) between Butantan and control groups. More neonates in the Butantan group required supplemental oxygen on day 28 of life (57 vs. 45%, *p* = 0.05). Neonates in the Butantan group also required more doses of surfactant (2.0 ± 0.8 vs. 1.5 ± 0.7; *p* < 0.001) and had a significantly higher incidence of pulmonary interstitial emphysema and PDA compared with controls.

In our study, a significantly higher number of GLSE infants (65%) required repeat doses of surfactant compared with 17% in the beractant group. In general, about a third of infants with RDS may require additional surfactant doses due to sub-optimal response to first dose [11, 12]. However, this number can increase to 50% if RDS is severe or additional complicating factors like pulmonary air leak, asphyxia, sepsis, or PDA are noted [13]. In preterm neonates with clinical presentation of respiratory distress, differentiating RDS (due to surfactant deficiency) from early-onset sepsis within a limited time frame is always a challenge. Since the incidence of sepsis and other complicating factors were comparable in the two groups, the poor efficacy of GLSE could be attributed to the product itself rather than due to host factors.

Despite being safe and efficacious in animal studies, efficacy of goat lung surfactant was poor when studied in human subjects in our trial. Potential reasons include differences in preparation, processing, or purification techniques, altered biophysical effects in human subjects, inadequate dosage, or increased susceptibility to inactivation by various bio-molecules in the host. Differences in surfactant composition may explain to a certain extent the variations in physio-structural and physiological properties noted in vivo at the alveolar air–liquid interface [14]. Poractant alfa, a minced porcine lung extract has a higher phospholipid content (76 mg/mL) compared with bovine lung preparations (25 mg/mL) [15]. It also has a higher content of plasmalogens, a component that improves the surface properties of phospholipid and surfactant protein B (SP-B). Recent work has shown that surfactants with low surface viscosity have better function as it allows a more homogeneous distribution of densely packed lipids during expiration [11]. Minor surfactant components like plasmalogens and polyunsaturated fatty acid containing phospholipids play a crucial role in minimizing surface viscosity. Although it is unclear if these physico-chemical variations in surfactant translate into clinically relevant outcomes in neonates, the understanding calls for a more in-depth analysis of the physio-structural properties of GLSE and whether addition of minor lipid components can enhance its surface-active properties [16].

A meta-analysis of randomized controlled trials comparing various animal-derived surfactants showed reduced risk of mortality, need for repeat doses of surfactant, PDA requiring treatment, and the composite outcome of death or BPD with poractant, compared with bovine surfactants [17]. These benefits were noted when a higher initial dose (200 mg/kg) of poractant was used than 100 mg/kg of either poractant or bovine surfactant. These findings question whether the poor efficacy of GLSE could be explained by a lower bio-equivalence of its dose compared with beractant. However, it is not clear if GLSE would have performed better at a higher initial dose. Such a preparation would have to be sufficiently concentrated that the intra-tracheally administered volume does not exceed 4–5 mL/kg. Higher dose of poractant (200 mg/kg) can be administered in a small volume of 2.5 mL/kg, compared with 3 or 4 mL/kg to administer 100 mg/kg of calfactant or beractant, respectively. Smaller volume of poractant facilitates quicker administration with fewer incidences of surfactant reflux and has definite cost savings from lower administration cost, less drug wastage, and reduced need for re-dosing [18]. At present, there are no dose equivalent trials comparing different animal-derived surfactants, and further research is needed.

Our study has several strengths. It is one of the rare examples of a new drug developed and tested in neonatal population with close collaboration between academia and industry in an LMIC setting. Despite the constraints of LMIC setting, we rigorously followed GCP. The trial was overseen by an independent DSMB, which provided a close oversight that culminated in the timely termination of the trial when concerns regarding lack of efficacy of GLSE emerged. The use of open-label beractant as a repeat dose, in both the groups, kept safety of the neonates above everything else, and was highly commended by the DSMB. The antenatal steroid use in the study population was high and all the study centers followed a uniform protocol for surfactant administration and re-dosing. The first dose was administered relatively early at a median age of 2.4 h in both the groups. We used an innovative system that ensured blinding of clinicians, in addition to investigators, parents, data teams, and statisticians. The study has some limitations. RDS was diagnosed clinically without the need for radiological confirmation. Hence, the possibility of the diagnosis being contaminated by other pulmonary conditions like pneumonia or asphyxia cannot be ruled out. The neonates were only followed until 36 weeks PMA due to logistic reasons, and long term outcomes were not studied.

In summary, in a multi-center, randomized controlled trial, we found that goat lung surfactant was inferior to beractant for the treatment of RDS in preterm neonates. Reasons to ascertain inferior efficacy of goat lung surfactant requires investigation and possible mitigating strategies in order to develop a low-cost and effective surfactant.

## Supplementary information

Supplementary Information

## Members of various committees involved in the GLSE trial

**International Advisory Board (IAB)**: Prof MK Bhan, Prof Anand Pandit, Prof Vinod Bhutani, Prof Haresh Kripalani, Prof Betty Kirkwood, Prof Neerja Bhatla, Dr Monika Bahl

**Research Steering Group (RSG)**: Prof Vinod Paul, Prof AK Deorari, Dr Nita Bhandari, Mr AB Ramteke, Prof Siddharth Ramji, Dr Shirshendu Mukherjee, Dr David Millar, Dr Nitya Wadhwa, Dr Bethan Hughes, Dr Diana Tay, Dr GB Nair, Dr Sudhakar Bangera

**Data and Safety Monitoring Board (DSMB)**: Dr Rajiv Bahl, Prof Allan Donner, Prof Ravinder Kaur, Dr Shinjini Bhatnagar, Dr Jose Carlos Martinez, Prof Rakesh Lodha, Prof SN Dwivedi, Prof Harish Chellani, Dr Pawandeep Kaur Dhawan

**Expert Committee on formulation of guidelines on the causality assessment team:** Prof YK Gupta, Prof AP Dubey, Dr Annam Visala, Prof Siddarth Ramji, Prof Vinod K Paul, Dr Vijay Prakash Mathur, Dr Sudhakar Bangera, Prof Ramesh Agarwal

**CDSA team**: Dr Nitya Wadhwa, Shantala Ponemone, Arun Kumar B Ramteke, Vimaljeet Kaur, Ranjan Kumar, Ligi Anil, Madhvi Sharma, Abhishek Anand, Sonali Gupta, Prashant Bhujbal, Sanjeeva Kumar, Arti Shandilya

**Wellcome:** Dr Shirshendu Mukherjee, Diana Tay, Bethan Hughes, Angela Miller
