# Supplementary material for: Goat lung surfactant for treatment of respiratory distress syndrome among preterm neonates: a multi-site randomized non-inferiority trial
Source: J Perinatol. 2019 Sep 4;39(Suppl 1):3–12. doi: 10.1038/s41372-019-0472-0 (PMC8075884; doi:10.1038/s41372-019-0472-0)
Supplement: Supplementary file 1 — Supplementary Information [file 41372_2019_472_MOESM1_ESM.docx]

**Supplementary Information for**

**“Goat lung surfactant for treatment of respiratory distress syndrome among preterm neonates: a multisite randomized non-inferiority trial”**

**Index**

Standard Operating Procedures (SOP) for Blinding

**Additional tables**

Table S1: List of Outcomes and their definitions

Table S2: Recruitment status at different sites

Table S3: Reasons for screen failures

Table S4: Type of respiratory support at investigative product (IP) administration: Eligibility criteria fulfilled

Table S5: Other study outcomes

Table S6: Details of deaths

**Standard Operating Procedures (SOP) for Blinding**

## **Purpose**

To ensure blinding of parents, clinical staff and research staff from the type of IP (Investigational product) administered

**Items provided**

1. Blinding Kit: contains

Sterile sheet (1m X 1m) - 1

Gloves (Size 7) - 2 pairs

Surgical blade - 1

Opaque syringe (10 mL) - 1

Needle size 21G - 1

Opaque catheter (Lactroguard®) - 1

One packs of sterile cotton gauzes (10 X 10 cm with a thickness of 2-3 mm)

Retrieval kit (white colored polybag for packing items used in the process of IP administration (IP vial, its cap, product insert, IP outer pack, syringe, needle and the catheter

2. The IP as per random sequence

3. Cloth screen/separate room

**Baseline preparation**

1. Identify an IP corner in the NICU

1. Identify a separate room in the vicinity of NICU where IP person can load IP in a syringe in the blinded fashion.
2. If room is not available, an isolated corner in the NICU can be identified. Keep a cloth screen available to cordon off this corner from rest of the NICU. Label the cloth screen as “FOR STUDY PURPOSE. DO NOT REMOVE FROM THIS AREA” using red fonts.
3. Always keep this place free from clutter.
4. There should be a platform for loading IP.
5. Keep a small clip board/white board to depict IP dose

2. Identify a team of IP Persons

1. Identify a team of nurses/residents (6 to 10) to play the role of IP person from units/wards other than NICU. IP loading person should NOT be from the NICU or from the research team. IP Persons will be duly acknowledged in the publications arising out of the trial.
2. Provide appropriate training to the IP persons for loading IP as per the SOP.
3. Maintain a list of their mobile no/internal no with their duty roaster.
4. Research nurse (RN) should always identify an IP person available in that shift.

3. The job description of IP person

1. Under aseptic procedure, load the requisite amount of IP in the opaque syringe
2. Attach the catheter to the syringe and do not cut and do not flush the catheter with IP.

**Before IP administration**

4. Research nurse (RN)

1. Call the IP Person
2. Make sure that IP corner is in order and free from any clutter
3. Cordon off IP corner with the cloth screen. Activities in IP corner must not be visible to people outside including RN
4. Calculate the dose of IP from the dosing chart (4 mL/kg; rounded off to next highest 0.5 mL). Write the dose on a piece of a paper and clip it on the clipboard at IP corner
5. Take out IP vial (each vial has three labels on it) as per the random strata. Stick one label on the CRF, second on the IP administration log and let the third label be on the IP vial itself.
6. Carry the IP vial box and one Masking Kit to the IP corner
7. Wash hands, open the Masking Kit, and spread the sterile sheet holding its edges on the table in IP corner.
8. Open the packaging of both the pairs of gloves, surgical blade, opaque syringe, needle, feeding tube and cotton swab and drop them on the sheet without touching any of the items or the sheet.
9. Soak the cotton swab with liberal amount of isopropyl alcohol
10. Fill the ID detail of infant on the retrieval kit.
11. Reads out the IP instruction sheet to the IP person. Brief the IP Person about the process of loading of IP and re-packaging of all items. The IP person has to take utmost care to maintain the blinding.
12. Once done, the RN leaves the IP closed corner/room and allows the IP person alone to remain in the closed corner/room

5. IP Person

1. Wash hands.
2. Checks if the door is closed or the screen is in place
3. Follows IP instruction sheet
4. Wear the first pair of gloves and the roll IP vial between palms to bring it to room temperature.
5. Wipe the vial with soaked cotton swab (provided) and place it on the sterile sheet.
6. Discard the first pair of gloves. Wear the second pair of gloves. Keep inner sterile wrapper of the gloves safely.
7. Open the IP vial and withdraw the required amount. Do not discard the vial or lid.
8. Place the loaded syringe with catheter blade in the sterile glove wrapper
9. Place the vial and its lid in the retrieval kit.
10. Now, go to the baby bed and hand over the wrapper containing loaded syringe and attached catheter to the clinical team at the bedside

6. Clinical team

1. RN reads out instructions to the clinical team (IP administration instruction sheet provided)
2. Clinical team trim the catheter to the appropriate length. Do not flush the catheter with IP.
3. Administers IP in four aliquots. After the last aliquot, flush the catheter with 1 mL air to ensure all the drug is delivered to the baby.

**During IP administration**

1. RN fills the details of section I of CRF
2. Keep an eye that no one goes to IP corner

**After IP administration**

IP Person will:

● Collect the used syringe and catheter from the clinical team

● Return back to IP corner behind the cloth screen or closed door

● Place the used vial of IP and its cap, package insert, outer box, used syringe and the used catheter in the retrieval kit. The used needle can be destroyed using local waste disposal policy.

● Complete the checklist on the retrieval kit

● Seal the kit and hand it over to RN.

Research nurse will keep sealed retrieval kit in her safe custody. This has to be handed over to study monitor on her/his next site visit.

**Additional tables**

**Table S1: List of Outcomes and their definitions**

| **Outcome** | **Definition** | **Outcome measurement** |
| --- | --- | --- |
| ***Primary*** | | |
| BPD free survival | Survival free from BPD defined as per the definition provided by NICHD | All the neonates were followed up round the clock by dedicated, in-house research staff for the development of BPD or death. |
| Need for repeat dose | Repeat dose, if required, always open-labeled Beractant in the dose same as the first dose (100 mg/kg).  Indication for repeat dose: FiO_2_ of ≥40% | A maximum of 3 repeat doses after 4-6 hours of giving previous dose or as per clinician discretion |
| ***Secondary*** | | |
| Area under the curve for (AUC) for oxygen requirement (FiO2) in first 48 h | The oxygen requirement was recorded every hour using a standardized protocol and AUC for first 48 hrs was calculated | The outcome was recorded by the research nurse and AUC calculated. |
| Pulmonary hemorrhage | Occurrence of bleeding/blood stained secretions from the trachea within 48 hr of surfactant administration | Monitoring of infants |
| Any air leak within 72 hours of administration of surfactant | Occurrence of any air leak -pneumothorax, PIE or pneumomediastinum in chest X-ray (done when clinically suspected) | A radiologist blinded to clinical data would report the X-ray. |
| IVH grade 3 or 4 | Grade 3 or 4 IVH as per Papile/Volpe classification | Ultrasonography was carried out by a radiologist/neonatologist at 72±24 h |
| PVL-cystic and non-cystic | PVL as per deVries classification | Ultrasonography was carried out by a radiologist/neonatologist at 72±24 hrs. 28±7 days |
| Sepsis | Occurrence of any episode of sepsis (culture positive or culture negative) from the time of enrolment until 7 days of age | Clinical monitoring of infant and investigation for sepsis as per standard protocol |
| Respiratory support at 72 h and 7 days of age | Mechanical ventilation/CPAP/HFNC/free flow oxygen/none at the two time points (point assessments) | Monitoring of infant using standard checklist |
| Retinopathy of prematurity (ROP) | ROP requiring laser as per ICROP classification | Eyes of the babies were examined by the ophthalmologist starting from 4 wks. of life |
| Neonatal mortality | Death of a neonate in first 28 days of life | Monitoring of infants |
| Duration of mechanical ventilation and CPAP | Cumulative duration of mechanical ventilation and CPAP until discharge/death | Monitoring of infants |
| Duration of hospital stay | Duration of hospital stay | Monitoring of infants |

**Table S2: Recruitment status at different sites**

| **Sites** | **Group** | | **Total**  **N (%)** |
| --- | --- | --- | --- |
|  | **GLSE N (%)** | **Beractant N (%)** |  |
| 1 | 7 (13.5) | 7 (15.2) | 14(14.3) |
| 2 | 10 (19.2) | 10 (21.7) | 20 (20.4) |
| 3 | 20 (38.5) | 18 (39.1) | 38(38.8) |
| 4 | 6 (11.5) | 3 (6.5) | 9(9.2) |
| 5 | 9 (17.3) | 8 (17.4) | 17(17.3) |
|  | 52 (100.0) | 46 (100.0) | 98 (100.0) |

*Note: 3 additional neonates were enrolled at 2 outborn sites in phase I of the study*

**Table S3: Reasons for screen failures**

| **Reason for screen failure** | **N (%)** |
| --- | --- |
| **Exclusion Criteria:**  Severe birth asphyxia (cord blood pH<7.0)  Malformation  Hydrops  Prophylactic surfactant  Pulmonary air leak  Shock  Pulmonary hemorrhage | 43 (15.8%)  36 (13.2%)  3 (1.1%)  10 (3.7%)  1 (0.4%)  8 (2.9%)  0 |
| **Other reasons:**  HIV positive  Weight >2 kg  LAMA / Charges  Death prior to enrolment | 1 (0.3%)  8 (2.9%)  15 (5.5%)  6 (2.2%) |
| Consent denied | 43 (15.8%) |
| Randomized | 98 (36.0%) |
| **Total** | 272 (100.0%) |

**Table S4: Type of respiratory support at investigative product (IP) administration: Eligibility criteria fulfilled**

| **Characteristic** | **GLSE**  (n=52) | **Beractant**  (n=46) | **p-value** |
| --- | --- | --- | --- |
| FiOFiO_2_ requirement 40% or more while the baby is on CPAP to maintain pre-ductal oxygen saturations 90%-95% | 41 (78.8) | 38 (82.6) | 0.638 |
| Nee Needing intubation because of CPAP failure or severe respiratory distress | 11 (21.2) | 8 (17.4) |  |

*Data expressed as n (%)*

**Table S5: Other study outcomes**

| **Outcomes** | **GLSE**  **(n=52)** | **Beractant**  **(n=46)** | **p-value** |
| --- | --- | --- | --- |
| Number of infants receiving mechanical ventilation at different time points after IP administration  At 1 hour  At 2 hours  At 12 hours  At 24 hours  At 48 hours  At 7 days  At 14 days  At 28 days  At 36 weeks of PMA | 26 (50)  27 (51.9)  34/50 (68.0)  32/49 (65.3)  26/42 (61.9)  15/36 (41.7)  6/33 (18.2)  3/32 (9.4)  0/31 | 16 (34.8)  17 (37.0)  15 (32.6)  15 (32.6)  15/44 (34.1)  10/39 (25.6)  5/36 (13.9)  5/35 (14.3)  3/33 (9.1) | 0.13  0.14  0.001  0.004  0.08  0.14  0.63  0.54  0.08 |
| Cumulative duration (h) of free flow oxygen  Mean ± SD  Median (IQR) | 143.5 ± 266  0 (0, 191) | 52.3 ± 121.9  0 (0, 14) | 0.036  0.26 |
| Cumulative duration (h) of CPAP, HFNC or NIV  Mean ± SD  Median (IQR) | 218 ± 296.7  135.5 (1,257) | 274.9 ± 367.1  121 (27,366) | 0.40  0.48 |
| Cumulative duration (h) of mechanical ventilation  Mean ± SD  Median (IQR) | 92.8 ± 122.4  44.5 (6.5,132.5) | 110.0 ± 233.5  20 (0,121) | 0.64  0.09 |
| Cumulative duration (h) of any respiratory support  Mean ± SD  Median (IQR) | 455.8 ± 457.6  295.5 (70.5,734.5) | 437.8 ± 486.2  244 (113,672) | 0.85  0.85 |

* Anytime during study time (from IP administration to 36 weeks PMA or death, whichever is earlier)

**Table S6: Details of deaths**

| **Deaths** | **GLSE**  (n=21) | **Beractant**  (n=14) | **p-value** |
| --- | --- | --- | --- |
| Single most important cause of death  Prematurity related complications  (includes intraventricular hemorrhage, pulmonary hemorrhage and severe respiratory distress due to extreme prematurity)  Sepsis  Others | 10 (47.6)  11 (52.4)  0 | 3 (21.4)  10 (71.4)  1(7.1) |  |
| Age at death (days)  Median (IQR) | 5.5 ± 6.9  3 (2,5) | 13.9 ± 16.2  8.5 (2, 17) | 0.04 |
| Deaths by gestation groups  26-27 wks  28-32 wks | 7/12 (58.3)  14/40 (35.0) | 4/8 (50.0)  10/38 (26.3) | 0.71  0.41 |
| Death by antenatal steroids groups  Incomplete course  Complete course  None | 6/17 (35.3)  12/27 (444)  3/8 (37.5) | 6/18 (33.3)  6/20 (30.0)  2/8 (25.0) | 0.90  0.31  0.59 |
| Death by interval between eligibility and IP administration  <1 hr  1 hr to <2 hr  2 hr to <6 hr  6 hr and more | 8/22 (36.4)  6/21 (28.6)  6/8 (75.0)  1/1 | 5/26 (19.2)  4/13 (30.8)  4/6 (66.7)  1/1 | 0.18  0.89  0.73 |
| Death by study site  1  2  3  4  5 | 3/7(42.9)  8/10 (80.0)  3/20 (15.0)  5/6 (83.3)  2/9 (22.2) | 0/7  5/10 (50.0)  4/18 (22.2)  2/3 (66.7)  3/8 (37.5) | 0.16  0.57  0.57  0.49 |
